# Phase-shifted imaging on multi-directional induction thermography

**DOI:** 10.1038/s41598-023-44363-5

**Published:** 2023-10-16

**Authors:** Benat Urtasun, Imanol Andonegui, Eider Gorostegui-Colinas

**Affiliations:** 1 LORTEK, Basque Research and Technology Alliance (BRTA), Arronamendia kalea 5A, 20240 Ordizia, Spain; 2 Robotics and Automation Group, Electronic and Computer Science Department, Faculty of Engineering, Mondragon University, Loramendi Kalea, 4, 20500 Arrasate-Mondragon, Spain

**Keywords:** Electrical and electronic engineering, Applied physics

## Abstract

A novel multi-directional eddy current thermography (ECT) system is presented generating sets of directional phase images that have been fused with a processing pipeline allowing for an improved probability of detection (POD). Inhomogeneous electromagnetic Joule heating derived from the diversion of induced eddy currents provoked by cracks, altering its path around as well as under its bottom, is the principal phenomenon enabling its usage as a non-destructive-evaluation (NDE) technique. Most induction thermography systems employ inductors derived from old designs, optimized for localized heating with a fixed magnetic field direction. This provokes a directional detection blind-spot for surfaces with random crack orientations. In this paper we have observed that the pattern associated with the thermal response distribution can be geometrically correlated to the relative orientation of the magnetic field regarding the crack, conforming to a rotating feature that has not been described before. Extracting the apparent motion as an optical flow, with a phase-shifting interpolation of the intermediate orientations, generates a signal that enables a robust segmentation of a wide variety of defects in ferritic and austenitic alloys. Its performance has been evaluated with two ‘Hit/Miss’ POD studies TIG welds Inconel 718 and Haynes 282 alloys. Results show an increased detectability regarding the manual labelling of the defects in the same directional set, employing the same input.

## Introduction

Industrial manufacturing processes such as welding, steel hot rolling or casting are prone to cracks and porosity defects. Yet these defective features act as a stress concentration site which negatively affect the fatigue life and tensile properties of as-built parts. Furthermore, when these faulty parts are subjected to large mechanical forces defects in these components could lead to fatal consequences. Therefore, it is crucial to identify these defective parts through a quality inspection process, on the one hand, to ensure product quality and, on the other hand, to enhance material circularity, requalifying it or reusing it in some other process, i.e., minimizing environmental footprint. The detection of cracks in manufactured metal alloys is conventionally performed with techniques such as the magnetic particle inspection (MPI) and fluorescent penetrant inspection (FPI).

However, the detectability of cracks using MPI and FPI is limited and depends on the inspector’s skill, which reduces the reliability of these techniques. Additionally, these methods require the use of toxic chemicals and may require the removal of the component’s coating before inspection, which increases both the manufacturing and inspection times. Moreover, the manual and time-consuming nature of traditional inspection processes does not fit well with modern manufacturing lines. As manufacturing processes have become increasingly automated and faster, inspection processes must keep pace to avoid creating bottlenecks in the production line. In industrial practice, both eddy current and induction thermography (IT)^1^, techniques are commonly used for 100 %-testing of components and for spot checks, though eddy current testing is limited to relatively simple geometries, fails to find large-scale defects as well as shallow inclined cracks, and does not allow to identify the cracks in thin materials. In this contribution, IT is featured as an interesting alternative to overcome these limitations.

IT uses a probe comprised of a coiled conductive piece of wire close to a test surface to generate eddy currents. In essence, exciting a time varying current on the coil to generate a magnetic field. In the proximity to the test surface, the magnetic field interacts with the test part inducing eddy currents running opposite to the currents in the probe. The phase and amplitude of these eddy currents depend on the conductivity, permeability, and homogeneity properties of the test part. Indeed, the non-zero resistivity of the material produces Joule heating radiation and in particular, test-parts discontinuities, such as cracks produce a noticeable change in the radiated heating distribution. The losses associated with hysteresis during induction heating have a measurable effect on ferromagnetic materials, as they result from the friction produced by the oscillation of the magnetic poles. However, the relative contribution of the hysteresis losses to the overall heating is comparatively small compared to the Joule heating losses associated with the induced eddy currents.

There is already a large literature focused on identifying the optimum parameter definition in the IT technique, i.e. finding the most suitable lock-in regimes considering the skin depth, the thermal penetration depth, and the crack dimensions to be detected^2–4^. These studies are mostly based on extensive empirical analysis from both laboratory and field investigations of faults and advanced diagnosis applications.

Undoubtedly, the results and conclusions of such investigations are of particular interest for current induction thermographic inspection applications, however, the detection of these cracks is strongly conditioned by the relative orientation between the crack and the direction of the magnetic field produced by the inductor, which is indeed a non-predictable variable.

Using a customary inductor, such as a dipole or a pair of Helmholtz coils which are composed of two coils, produces a fixed magnetic field with a predominant direction. This limits the resulting thermal disparity in the vicinity of the cracks in certain configurations. The magnitude of this effect is minimized when both the direction of the crack and the resulting eddy currents associated to the magnetic field are aligned^3,5,6^. The robust detection of the cracks, regardless of its orientation is indeed one of the main challenges of IT. There is though a very limited number of studies dedicated to overcoming this critical drawback in IT, indeed the majority of modern induction systems stem from functional old designs that have been subsequently optimized to enhance induction heating, have been inherited from welding and thermal treatment applications^7,8^, and thus the generated magnetic field shows a unique direction in most cases. This limitation of the inductor can be overcome by scanning the area considering different orientations relative to the inductor. This rotation would require an external intervention such as a robot gripper but it also increases the inspection time, its cost and makes it more complex. Angle-independent thermo-inductive system have been previously demonstrated, employing a pair of Helmholtz coils disposed in an orthogonal manner containing the inspected part, resulting in a varying magnetic field orientation derived from the vector addition associated to an induction frequency differential^9^. The required containment of the part limits the potential use-cases of the system to small parts. Other approaches have recurred to the circular polarization of the eddy currents employing a tetra-pole inductor^10,11^ which generates a rotating magnetic field.

The main contributions of this paper lie in: (1) the identification of a new type of rotational feature associated to the tips and narrow points of the cracks and defects, which can be geometrically correlated with the direction of the magnetic field; (2) the development of an interpolation method to approximate the intermediate orientations of the magnetic field, while canceling a large part of the noise present in the phase images, which is not affected by the variation of the orientation of the magnetic field. (3) The design of a processing algorithm that fuses the directional phase images of the thermography, on a pixel-wise basis, exploiting the apparent motion of the pattern associated to the cracks, which has demonstrated good results in different materials and defect types. (4)The rigorous evaluation of the performance for the automatic detection of the cracks, following the MIL-HDBK-1823A standard, in two distinct types of TIG weld defects on Inconel 718 and Haynes 282, with 218 and 337 cracks respectively. Obtaining the a90/95 crack size, that is the crack size that can be detected with 90% probability with a confidence level of 95%. The results demonstrate a probability of detection that is not dependent on the relative orientation, with an increased detectability regarding the manual identification of the cracks in their respective phase images.

## Methods

### A multi-directional induction thermography system

An inductive thermography inspection system consists of three main components: a wave generator, an infrared camera, and an inductor. During the inspection, the inductor, powered by an alternating current, generates a magnetic field. This magnetic field interacts with the material, inducing eddy currents beneath the surface. The density distribution of these currents shows an exponential decay as you move away from the surface, decreasing to 1/e (approximately 37%) at the skin depth, $\delta _{ec}$. The skin depth is determined by factors such as the material’s resistivity $\rho$, the frequency of the eddy currents *f*, and the vacuum $\mu _{0}$ and relative $\mu _{r}$ magnetic permeability, i.e. $\delta _{ec}=\sqrt{\rho / \left( \pi f \mu _{0}\mu _{r}\right) }$.

The skin effect has implications for thermographic inspections because it affects the depth at which thermal patterns are detected. Higher-frequency currents or fields have shallower skin depths, resulting in more surface-sensitive measurements. Conversely, lower-frequency currents or fields penetrate deeper into the material, allowing for the detection of thermal patterns at greater depths. Moreover, surface geometry, the isotropy of the material, and the presence of cracks, pores, and sharp edges strongly influence the distribution and path of the eddy currents and lead to localized Joule heating. This heating causes a localized increase of the emitted infrared radiation, which is typically captured and measured using an infrared camera. While this procedure is widely used, there remains a need for critical analysis of these measurements to accurately assess the presence or absence of defects. Interestingly, the design and configuration of the induction device significantly influence the distribution of the radiated field, thereby presenting additional challenges in the analysis process.

Conventional inductor designs, including dipoles and Helmholtz coils, are commonly constructed with twin or single coils that generate a magnetic field with a fixed direction. Consequently, the induced eddy currents exhibit a predominant direction aligned with the magnetic field. The interaction between the eddy currents and the defects is also influenced by the relative orientation of both, potentially causing the currents to flow around or beneath the defects. As previously stated, the presence of defects and the interplay with the eddy currents have been quantifiably observed in studies, particularly when the crack and the eddy currents are not parallel. Consequently, conventional inductors face significant limitations in consistently detecting defects in random crack samples. In contrast, a tunable induction system capable of rotating the induced magnetic field overcomes potential directional blind spots. A multidirectional inductor, capable of comprehensive coverage of all crack directions under examination, ensures that all cracks are subjected to the thermal stimulus. This capability becomes particularly important when dealing with complex shapes, irregular geometries, or components with varying thicknesses. With this regard, we devised a new tetra-polar inductor, based on the tetra-polar magnetic yoke reported in^10^, though it features the ability to further control the induced magnetic field on it.

**Figure 1:**
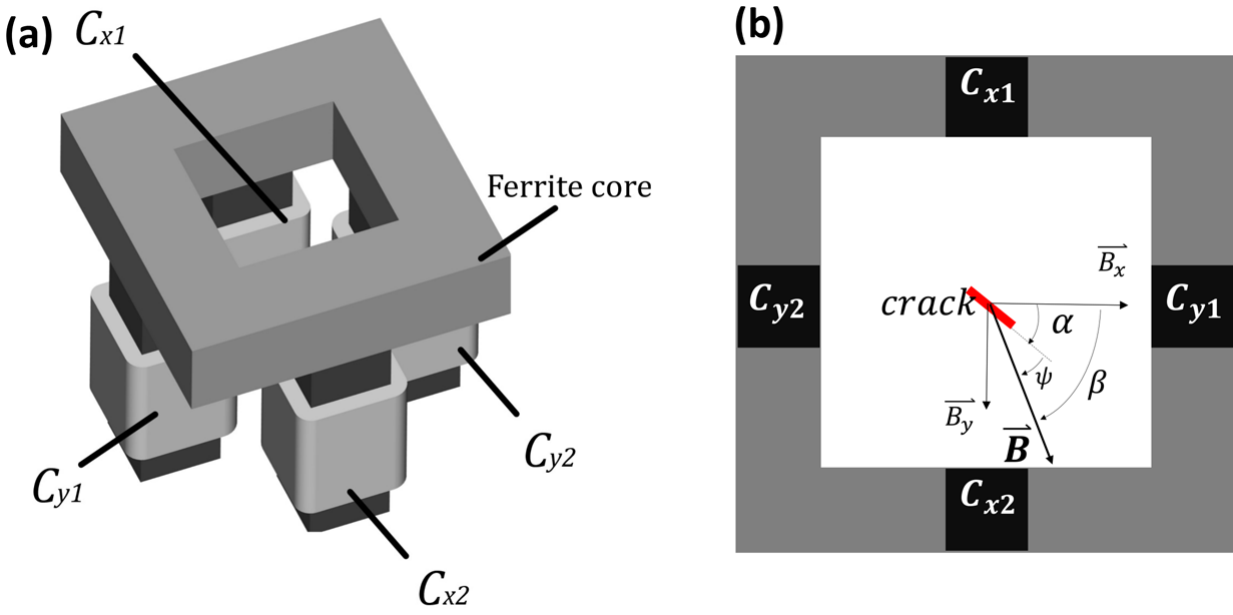
Tetra-pole inductor (**a**) a 3D illustration based on^10^, with 2 pair of independent coils and (**b**) is a top view of the inductor defining the relative orientation of the magnetic field, $\beta$, and the crack, $\alpha$, regarding the inductor, and the angle between both, $\psi$.

The inductor, shown in Fig. 1, is comprised of 2 pairs of coils, $C_{x}$ and $C_{y}$, arranged along the horizontal and vertical sides of a hollow square-shaped ferrite. This configuration allows for the convenient placement of an infrared camera on top, ensuring a direct line of sight and visibility to the inspected surface.

The resulting magnetic field of the 2 dipoles can be expressed as a vector addition in $\mathbb {R}^2$ as $\textbf{B}=\mathbf {B_{x}} sin(2 \pi f_{x} + \phi _{x} ) + \mathbf {B_{y}} sin(2\pi f_{y} + \phi _{y})$, where $B_{x}$, $B_{y}$, $f_x$, $f_y$, $\phi _{x}$ and $\phi _{y}$ are the field amplitudes, frequency and phase shifts on the transversal directions, respectively. Thus, by activating or deactivating the coils and reversing their polarity, the tetra-polar inductor can achieve four distinct orientations of the magnetic field, which we will hereafter denote as $\beta _{i}$, where *i* is one of the four orientations available using the proposed system. The specific orientations and their corresponding labels are presented in Table 1.

**Table 1:** Magnetic field orientations as a function of the activation of the coils.

$\beta$	State
$\beta _0$	$V_x \ne 0, V_y = 0$
$\beta _{45}$	$V_x = V_y \ne 0$
$\beta _{90}$	$V_x = 0, V_y \ne 0$
$\beta _{135}$	$V_x = -V_y \ne 0$

**Figure 2:**
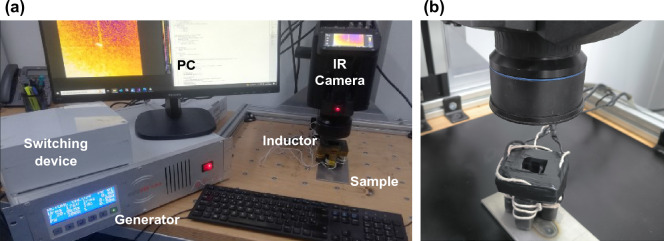
Multi-directional induction thermography system setup. (**a**) Overall system composed by an induction generator, switching box, PC, IR camera, tetra-pole inductor and a welded sample. (**b**) A closeup of the camera inductor and welded sample.

The complete experimental setup is shown in Fig. 2. It includes a Flir X6541sc infrared camera equipped with a Stirling-cooled 640x512 InSb sensor. This camera is capable of achieving frame rates of 125 Hz at full frame or 4 kHz in windowing mode. The induction generator utilized is the Edevis ITVis 3000 MHF, which has been employed in various scenarios and was developed in^12^. It incorporates a full-bridge inverter circuit with pulse-width modulation, operating within a continuous frequency range of 10 kHz to 60 kHz. And a switching device to alternate between the different states.

### Preliminary experiments on notches

To demonstrate the multi-directional induction capability of the system, a set of experiments has been carried out to analyze this aspect. Previous research has empirically demonstrated the relation between the thermal response and the relative orientation between the longitudinal direction of the crack and the magnetic field^3,5,6^.

This relation can be used to determine if the magnetic field is effectively rotating, by generating a similar thermal response by maintaining the same relative orientation of the magnetic field, $\beta$, and the crack, $\alpha$, that we denote $\psi = \alpha - \beta$, as shown in Fig. 1b. The thermal radiation is measured by the infrared sensor, which is affected by local emissivity variations, inhomogeneous initial surface temperature, as well as the directional emissivity, among others. The bias introduced by the raw thermal recording is mitigated by selecting the phase image of the carrier frequency of the pulses, associated to the Discrete Fourier Transform (DFT) of the whole thermal recording^1^. Note that in this case, the carrier frequency is typically called lock-in frequency. This results in the most accepted metric to evaluate the thermal response for this type of application, defined as the absolute phase contrast^13^, subtracting the local maxima and minima of the pattern, denoted as $\Delta \phi =\phi _{max}-\phi _{min}$. To simplify the analysis, two artificial notches have been produced with Electrical Discharge Machining, with 0.7 mm and 1.3 mm length and the same depth and width of 0.75 mm and 0.1 mm respectively. Note that the notched surface material is Inconel 718, in a sample measuring 4.75 x 80 x 150 mm. The set of experiments combines the 4 discrete orientations of the magnetic field, $\beta$, combined with 4 identical rotations of the crack, $\alpha$, resulting in 16 measurements for each notch.

**Table 2:** Experimental parameters describing the recording and thermo-inductive parameters associated to the preliminary test, probability of detection and supplementary samples.

Experiment	$f_{induction}$	*Framerate*	$t_{integration}$	PWM	$f_{lock-in}$	Duty ratio	Pulses per state	Resolution
	kHz	Hz	ms	$\%$	Hz	$\%$		mm/px
Preliminary test	20	300	1	50	5	50	3	0.75
POD	30	220	1	50	5	50	3	0.75
Forged Crankshaft	50	300	1	55	6	50	6	0.75
Steel billet	40	300	1	75	2.5	50	3	0.75
Forged Steel Bolt	40	300	1	70	2.5	50	3	1.00

**Figure 3:**
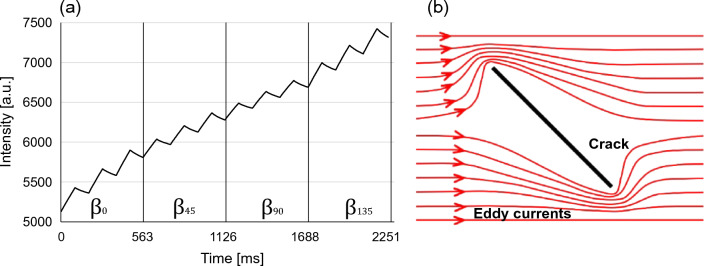
Multi-directional thermogram. (**a**) Raw IR thermogram of a sound area subjected to a 5 Hz multidirectional square wave induction with 3 pulses per direction, (**b**) Symbolic eddy current distribution circumventing oblique crack.

The measurements parameters are described in the first line of Table 2 describing the heating and cooling time of each pulse as a function of the lock-in frequency and duty ratio of a square wave. The switching occurs during the cooling of the third pulse of each $\beta$ state, enabling the continuous recording of the 4 states, as illustrated in Fig. 3a, showing a thermogram corresponding to a sound area.

**Figure 4:**
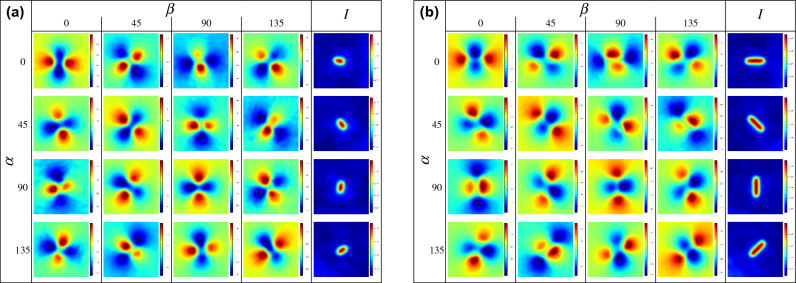
Phase images on notches, as a function of the magnetic field orientation. The first four columns shows the phase images in degrees as a function of the direction of $\beta$ and the last one corresponds to the amplitude of the FFT. The rows displays the notches in different directions, $\alpha$. (**a**) 0.7 mm, (**b**) 1.3 mm.

**Figure 5:**
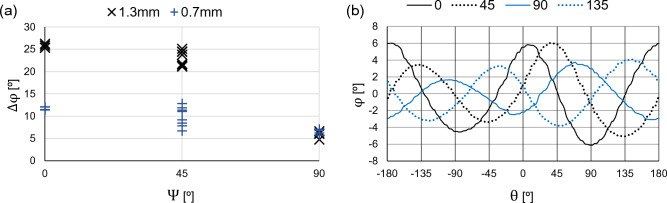
Directional Phase contrasts. (**a**) Phase Contrast as a function of the relative orientation between the the artificial defect and the magnetic field $\psi$, (**b**) Polar profiles of the phase images centered in the crack with a radius of 0.4 mm, coinciding with phase maximum of the 0.7 mm notch and $\alpha =0^{\circ }$.

Note that both the inductor and the camera remain stationary for all the measurements, with the same recording and thermo-inductive parameters. Figure 4 shows the experimental results, with the phase images as a function of $\alpha$ for each row, and $\beta$ for each column. The upper left diagonal phase images, with $\psi = 0^{\circ }$, exhibit the same rotated pattern. The last column shows the amplitude with the maximum increase of intensity perceived by the camera in arbitrary intensity units with $\psi = 0^{\circ }$, resulting in the highlighting of the notch. Note that the real cracks won’t produce such amplitude images as it will be shown later. The pattern exhibits a localized heat originating from the tips of the notch, associated to a higher eddy current density. Whereas the middle of the notch displays the opposite trend linked to the diversion eddy currents under the crack. Figure 5a displays the phase contrast of the whole set as a function of $\psi$, with a maximal phase contrast with $\psi = 0$. Note the phase contrast is directly correlated to *psi* confirming an equivalent thermal response associated to the direction of the magnetic field.

Another aspect to consider, that has not been previously exposed, is the apparent rotation of the pattern as a function of $\psi$. Figure 5b shows the phase image profiles of the 4 $\beta$ states with $\alpha =0$, in the 0.7 mm notch, plotting the phase profile of a 0.4mm radius semi-circle centered in the notch, with $\theta$ being the counterclockwise angle formed by each point, the center of the notch and the horizontal axis.

The resulting polar profile of each $\beta$, resembles a 2Hz sinusoidal wave with a $\beta$ phase-shift , that can be modelled with the following expression,

1 $$\begin{aligned} I(\theta ,\beta )=\mu (\theta )+\phi (\alpha ,\beta )cos(2\theta -\alpha -\beta ) \end{aligned}$$

with $\mu$ representing the average and $\phi$ the phase contrast as a function of $\alpha$ and $\beta$. Note that the apparent motion limits the precise estimation of the orientation and length of the crack, employing a single $\beta$. In this scenario, the apparent rotation of the pattern can be explained by the varying asymmetry of the eddy current distribution circumventing an oblique crack around its tips, as illustrated in in Fig. 3b.

### Thermo-inductive phase-shifting

The shifted polar profiles shown in Fig. 5b are similar to a wave form phase-shifting. As a result, Eq. (1), can be adapted to the fundamental phase-shifting equation, assuming that the global displacement of the signals is associated to the magnetic field orientation, $\beta$, with a phase, $\gamma$, the mean, $\mu$, the amplitude, $\phi$, and the pixel intensity of the $\beta$ signal, *I*, resulting in the following expression.

2 $$\begin{aligned} I(x,y,\beta )=\mu (x,y)+\phi (x,y)cos(\gamma (x,y)+\beta ) \end{aligned}$$

Many methods have been exposed to compute the pixel-wise phase-shifting with predefined shifting sequences^14,15^. In the current induction scheme shifting sequence, (0, 45, 90, 135), a generalized method, such as the one exposed in^16^ has been employed, yielding the global solution, as a function of the pixel intensities.

3 $$\begin{aligned} \gamma = {{\,\textrm{atan2}\,}}\left( \frac{I_{\beta _{45}} - I_{\beta _{135}}}{I_{\beta _{0}} - I_{\beta _{90} } } \right) \quad \quad \phi = \frac{1}{2} \sqrt{ (I_{\beta _{0} } - I_{\beta _{90}} )^2 + (I_{\beta _{45} } - I_{\beta _{135}} )^2 } \quad \quad \mu = \frac{1}{4} ( I_{\beta _{0}} + I_{\beta _{45}} + I_{\beta _{90}} + I_{\beta _{135}} ) \end{aligned}$$

**Figure 6:**
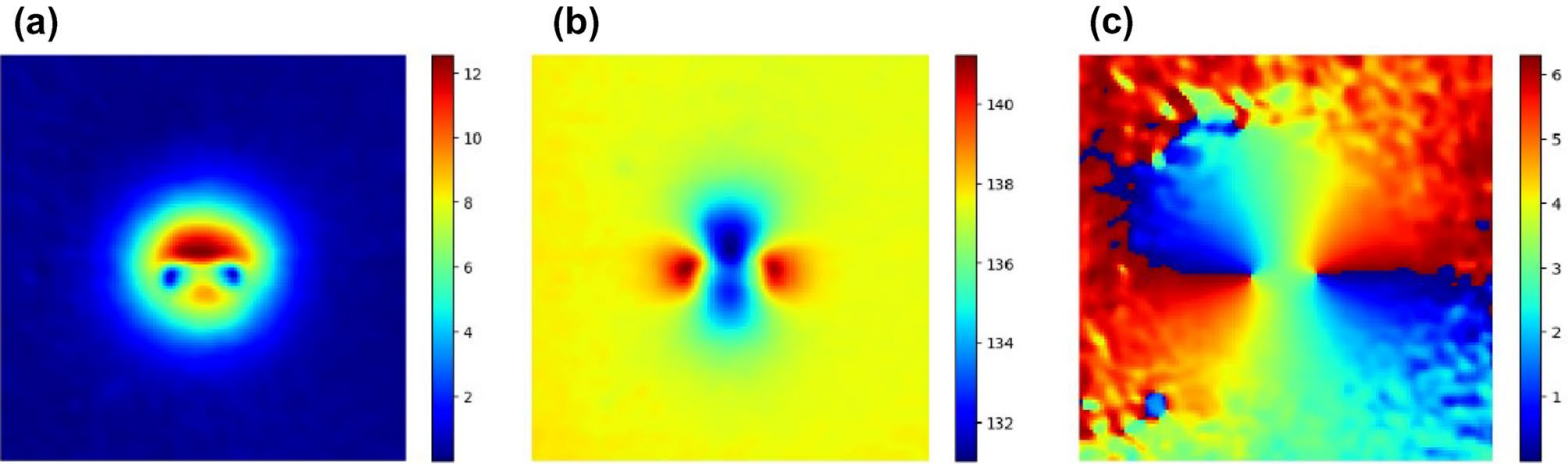
Thermo-inductive phase-shifting. Image corresponding to the parameters of the phase-shifting of the four phase images of the carrier frequency (**a**) $\phi$ amplitude [$^{\circ }$], (**b**) $\mu$ average [$^{\circ }$], (**c**) $\gamma$ phase-shift [rad].

The resulting phase-shifted images for the sequence of images for $\alpha =0$ are shown in Fig. 6.

**Figure 7:**
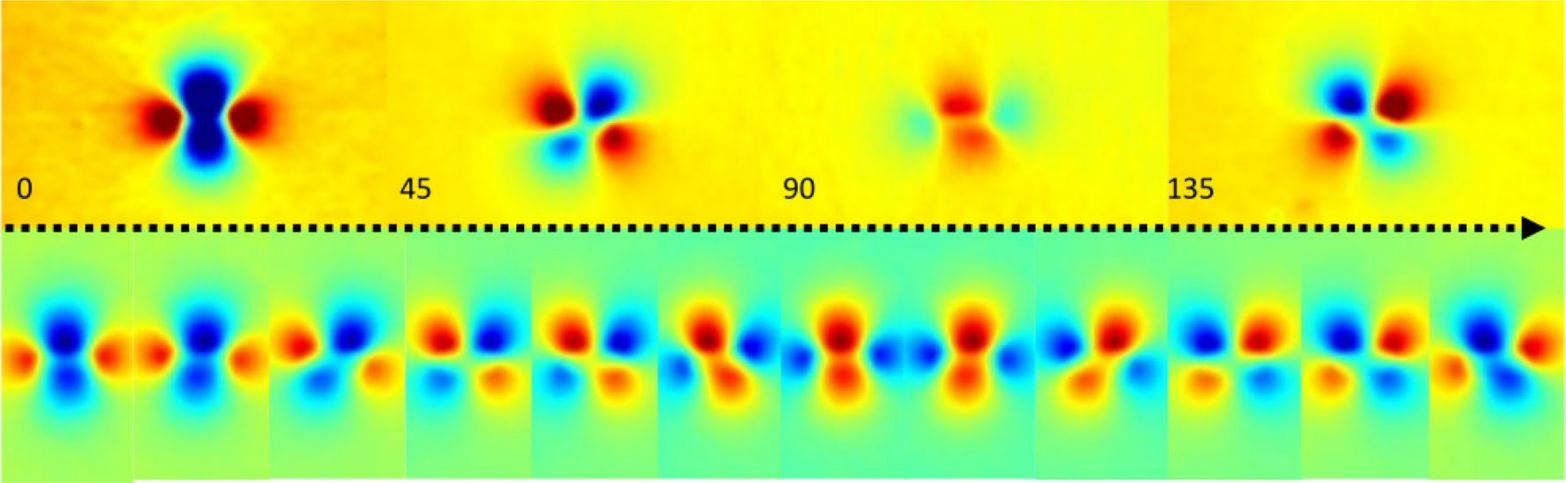
Normalized phase-shifted interpolation. Upper row with the 4 original phase images of the 1.3 mm notch with $\alpha =0$, below a sequence of synthetic phase-shifted images with a uniform sampling of $\beta$ covering the same range.

The amplitude, $\phi$ exhibits an increased signal associated to the pixel-wise variance of the four images, the average, $\mu$, is clearly correlated to the image spotting the highest amplitude and the phase-shift, $\gamma$, shows a concentric pattern centered in the notch, corresponding to a monotonic variation of the relative orientation of the crack. The resulting model can be effectively used to continuously sample the intermediate $\beta$ orientations with the expression (2). Another clear benefit is that the subtraction of the mean can reduce the noise. Figure 7 displays on the upper row the 4 original phase images of the 1.3 mm notch with $\alpha =0$, and below the uniformly sampled $\beta$ synthetic images corresponding to the same range subtracting the mean.

### Phase-shifted optical flow

The normalized rotational motion associated from the phase-shifted interpolation can be effectively described as an invariant feature of the cracks. The apparent motion between two images can be estimated with a method known as the optical flow (OF). The relative translation between two consecutive frames can be defined as $I(x,y,t)=I(x+\Delta x,y+\Delta y,t+ \Delta t)$, which can be extrapolated to a dense or sparse planar vector field defined on a bounded set $\mathbb {R}^2$, as $\Omega (x,y)=(\Delta x, \Delta y)^T = (u,v)^T$, with $u = \frac{\partial x}{\partial t}$, $v= \frac{\partial y}{\partial t}$. The OF estimation has been resolved in most of the classical OF algorithms recurring to correlation-based methods, such as the seminal work of Horn-Schunck^17^, which is based on two assumptions. The first one being a photometric consistency of the moving point formulated as a line constraint or direction, written as,

4 $$\begin{aligned} \frac{dI}{dt} = 0 = \frac{\partial I}{\partial x } \frac{\partial x}{\partial t} + \frac{\partial I}{\partial y}\frac{ \partial y}{\partial t} + \frac{\partial I}{\partial t} = E_x u + E_y v + E_t \end{aligned}$$

with $E_x$, $E_y$ and $E_t$ being the partial derivatives of the image. The second assumption is a term that enforces the smoothness of the solution by reducing the differences of the local flow, formulated as the minimization of the Laplacian of $\Omega$, written as: $\nabla ^2 u=\frac{\partial ^2 u}{\partial x^2}+\frac{\partial ^2 u}{\partial y^2}$ and $\nabla ^2 v=\frac{\partial ^2 v}{\partial x^2}+\frac{\partial ^2 v}{\partial y^2}$.

Other common methods, rely on image pyramids to extrapolate the flow regardless of the scale^18^, while modern approaches employ supervised Convolutional Neural Networks showing an improved performance^19^. OF thermographic use-cases have been mainly employed in image stabilization to generate pseudo-static sequences^20^, as well as an alternative thermal signal processing^21^.

**Figure 8:**
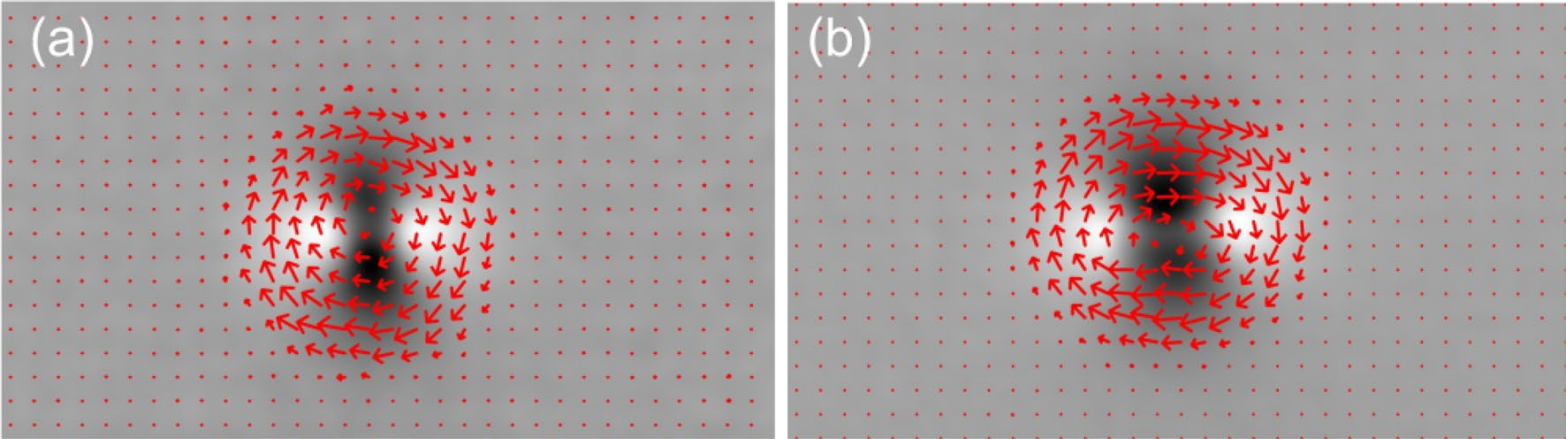
Phase-shifted optical flow average overlayed on top of the first phase image. (**a**) 0.7 mm, (**b**) 1.3 mm.

A set of flows have been generated employing the method exposed by Farnebäck^22^, implemented in OpenCV, computing the consecutive synthetic images uniformly sampled with the expression (2), subtracting the mean. Considering a rotational motion, the flow direction is a-priori constant which can be averaged to reduce the temporal noise. Figure 8 shows the average flow of 100 images uniformly sampled in the following cyclic range of $\beta$, [0, 45, 90, 135, 0), for both notches and $\alpha =0$.

### Rotation vector field convolution

A fundamental concept of vector calculus, employed in fluid dynamics and electrodynamics among other fields, known as the Helmholtz decomposition, provides a simple solution to the motion analysis of the flow. Based on the assumption that the fluids are typically modelled as irrotational and incompressible, a set of constraints can be stablished, leading to scalar functions associated to its velocity vector field. Therefore, the optical flow can be decomposed as the summation of two components $\Omega =\Omega _c+\Omega _r$, with $\Omega _c$, being the incompressible or divergent free component with $0=\nabla \cdot \Omega _c$, and $\Omega _r$ is the irrotational or curl free component with $0 = \nabla \times \Omega _r$. $\nabla$ denotes the symbolic operator whose components represent the partial derivatives, $\nabla = ( \frac{\partial }{\partial x},\frac{\partial }{\partial y})$. The spatial integral of $\nabla \cdot \Omega = \frac{\partial u}{\partial x } + \frac{\partial v}{\partial y}$ over a region is called the divergence, and its orthogonal complementary, $\nabla \times \Omega = \frac{\partial u}{\partial y}-\frac{\partial v}{\partial x}$, is its vorticity.

**Figure 9:**
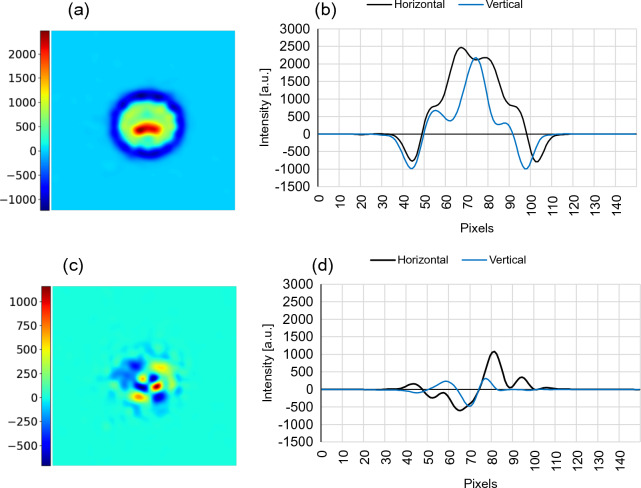
Motion field potential functions. (**a**) Intensity and orthogonal cross-sections of the vorticity (**a**,**b**), and the divergence (**c**,**d**).

As a result, the following expressions can approximate the vorticity and divergence, adapted to the optical flow,

5 $$\begin{aligned} w_{vort}=\sum _{i}^{m}{\sum _{j}^{n}\left| {\hat{r}}_{ij}\times \Omega _{ij}\right| \ }, \quad \quad w_{div}=\sum _{i}^{m}{\sum _{j}^{n}\left| {\hat{r}}_{ij}\cdot \Omega _{ij}\right| \ }\ \end{aligned}$$

with ${\hat{r}}_{ij}$ being the local unit vector. Figure 9a and b shows the vorticity and the orthogonal cross-sections, and Fig. 9c and d displays the divergence with its corresponding cross-sections.

The maximum vorticity is centered in the crack with a depression surrounding it below the background signal. On the other hand, the divergence does not exhibit a clear convergence to the center of the notch. Considering that the vorticity is correlated to the center of the notch, a simple blob detector implemented in OpenCV, can effectively estimate the location of the cracks. The results will employ the vorticity with a positive truncation.

## Results

### Experimental measurements on different materials

**Figure 10:**
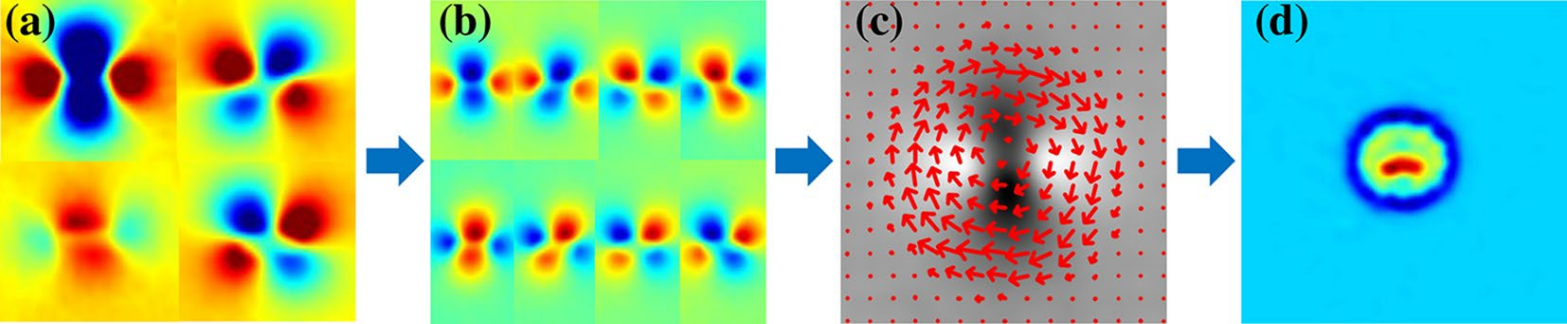
Phase-shifted induction thermography processing pipeline. (**a**) phase images of each direction, (**b**) phase-shifted uniform sampling, (**c**) optical flow motion, (**d**) vorticity of the vector field.

To evaluate the performance of the exposed methods, summarized in Fig. 10, a set of experiments has been carried out in different components made of different materials and with a diverse set of cracks.

**Figure 11:**
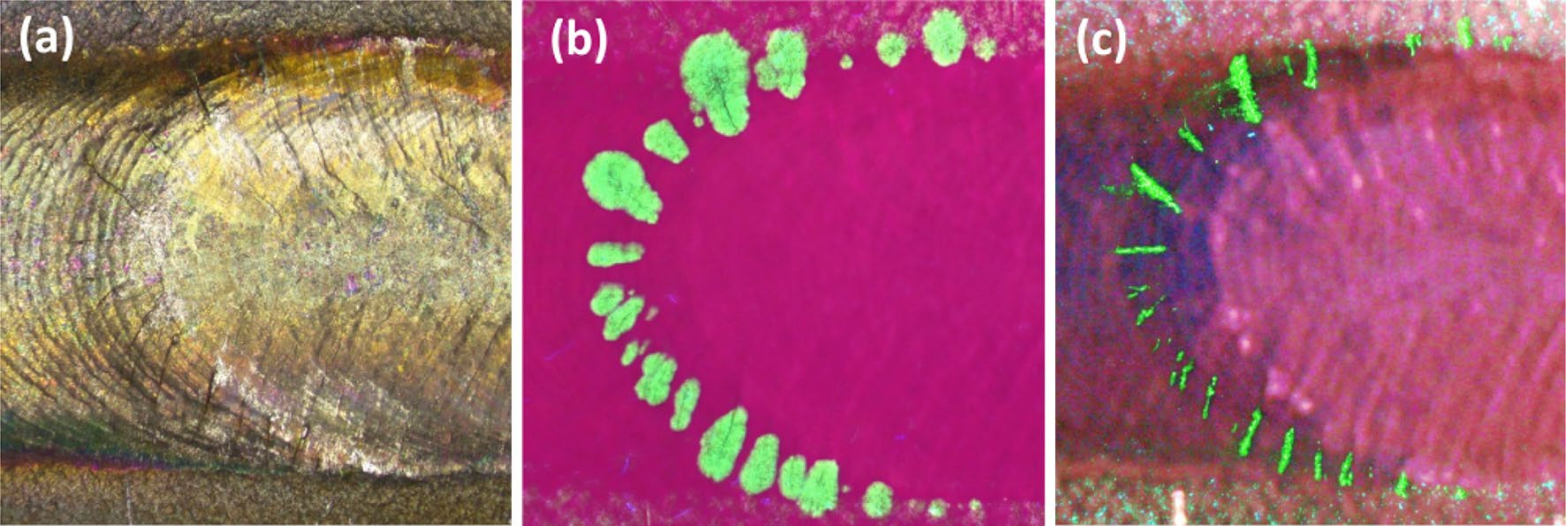
Modified fluorescent penetrant inspection TIG Inconel weld (**a**) clean surface, (**b**) Standard FPI under UV light after the application of a developer, (**c**) Enhanced FPI under UV light after removing the excess of the fluorescent liquid.

A set of samples with natural cracks arising from the TIG and Laser welding has been characterized with a different set of methods, to enable a subsequent comparison. First, standard FPI inspections have been carried out according to ISO 23277, by a certified inspector, leading to a partial detection of the cracks, as shown in Fig. 11a. This procedure involves, among other things, the application of a low-viscosity fluorescent penetrant liquid that impregnates the cavities of the cracks after a certain time defined by the standard, depending on the liquid viscosity, capillarity, as well as surface tension. Subsequent removal of the excess and the final application of a developer causes the remaining penetrant liquid to bleed to the surface, allowing an easier detection. Due to the proximity of the cracks, this last step provokes widespread staining of contiguous cracks, impeding its distinct identification. In other words, the standard FPI inspection does not have enough resolution to individualize and measure the cracks, as shown in Fig. 11b.

To enable a precise determination of the length and shape of the cracks, the standard procedure has been modified, by excluding the application of the final developer. Under a high intensity UV lamp, the samples are imaged with a 7 $\mu$m/px resolution Leica microscope, revealing the fluorescent liquid internally permeated in the crack, as shown in Fig.  11c. In a second step, macrographs have been taken with a Leica microscope (Leica DVM6), which has allowed for precise measurements of the length of the cracks (Fig. 11a) under the same frame.

**Figure 12:**
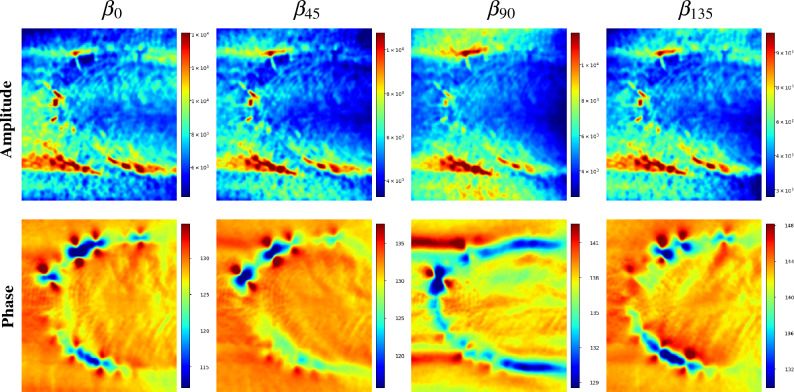
Multi-directional DFT in TIG Haynes weld. Upper and lower row displaying the directional amplitudes in arbitrary units and phases in degrees of the carrier frequency, associated to the four directions, $\beta$.

Figure 12 displays the multi-directional DFT amplitude and phase images of the sample shown in Fig. 11. As can be observed, the directional amplitudes, shown in the first row, do not exhibit clear differences that might increase the available information. Note that some of the isolated cracks can be easily identified but its pattern can be mistakenly associated to common features, such as the natural welding surface texture and superficial scratches, among other patterns. The second row displays the multi-directional phase images, exhibiting the shifted butterfly pattern with a varying overlap of the pattern near the clusters of parallel cracks.

**Figure 13:**
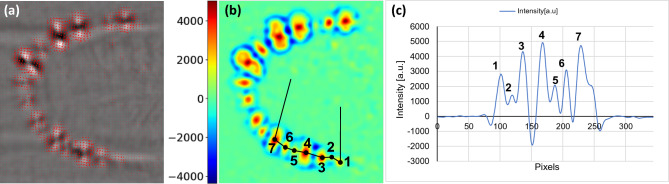
Phase-shifted optical flow vorticity signal profiles. (**a**) Optical flow of Haynes weld overlaid on top of the phase image, (**b**) Vorticity, (**c**) Vorticity profile highlighting the peaks corresponding to the center of the cracks.

Figure 13a shows the phase-shifted optical flow with an increased amplitude of the flow concentric to the cracks. Figure 13b displays the resulting vorticity with a cross-section traversing a cluster of parallel cracks, plotted in Fig. 13c. Note that the depression surrounding the cracks enables the distinct identification of the cracks.

**Figure 14:**
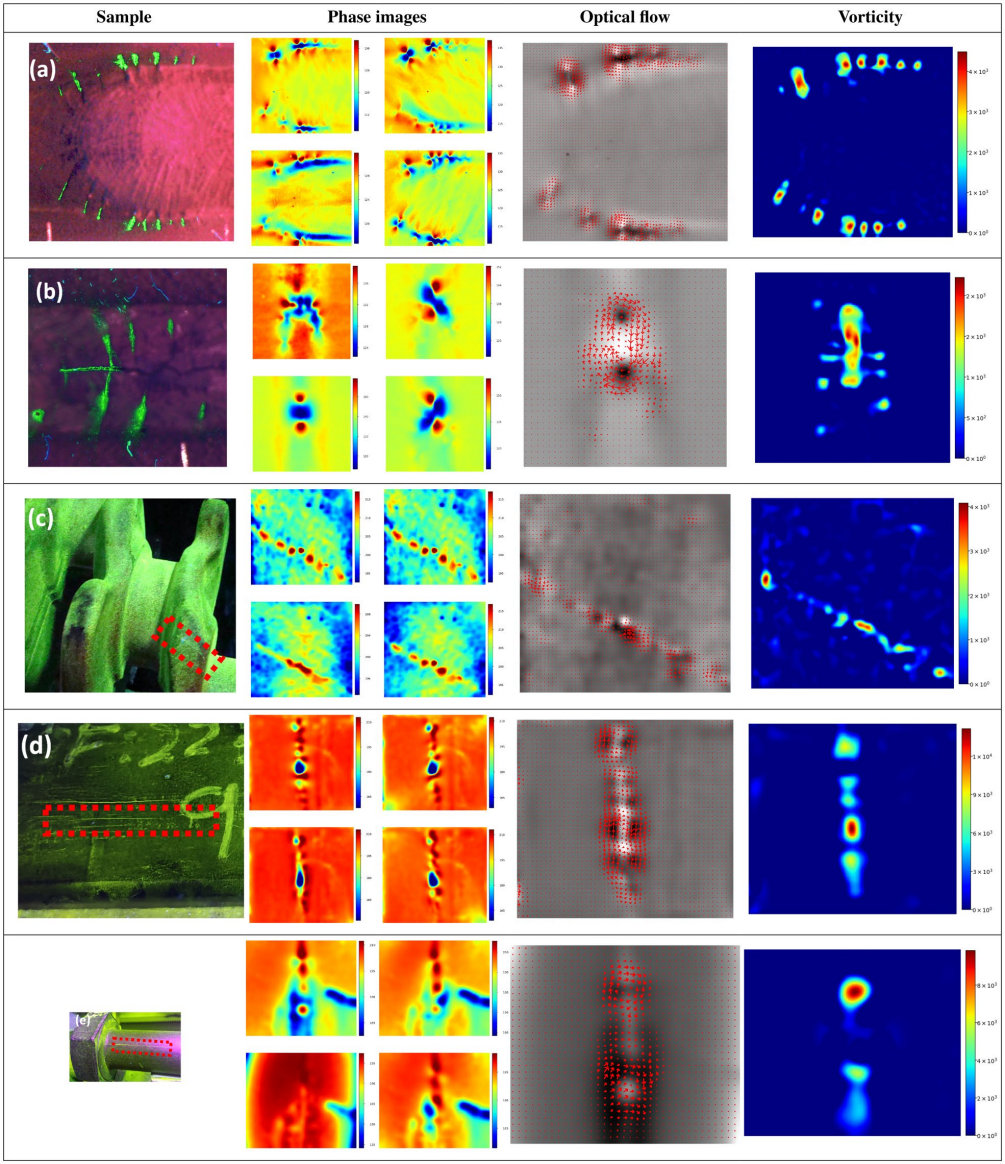
Natural weld crack detection. The first column displays the samples with the enhanced FPI for TIG inconel (**a**) and a laser inconel 718 (**b**), and a MPI for (**c**) forged steel crankshaft, (**d**) steel billet and (**e**) forged steel bolt. The remaining columns displays the multi-directional phase images, the optical flow, as well as the vorticity with a positive truncation.

Considering the positive results shown in the previous sample, the method has been tested in other samples, and materials with different types of cracks, as shown in Fig. 14, with the experimental parameters shown in Table 2, with the first two samples employing same values of the POD.

Note that Fig. 14a and b display a TIG and laser welds made on an Inconel 718 sample, with the modified FPI procedure in the first column. Figure 14a exhibits a set of cracks with a similar distribution as the one shown in Fig. 13, but Fig. 14b has the commonly known “fishbone crack” with a large crack centred along the weld and very small radial cracks. Figure 14c, d and e, shows a forged steel crankshaft, a steel billet, as well as a forged steel bolt respectively, with a MPI in the first column.

Note that the welded samples exhibit the typical butterfly pattern, resulting in a vorticity that clearly correlates the centre of the cracks. On the other hand, the steel samples have longer cracks, typically associated with lamination or forging defects producing a very different longitudinal pattern. However, its rotational component is clearly visible in the discontinuities and bridges of the cracks. This suggests that the narrow gaps and sharp discontinuities, such as ones present in the bridges of the cracks and the crack-tips, are locally constraining the direction of the eddy currents, regardless of the predominant magnetic field orientation, thereby altering the local eddy current density distribution.

### POD on Inconel 718 and Haynes 282 with TIG welds

In the previous section, a robust establishment of the ground-truth of the defects has been presented and all cracks have been measured. This characterization is necessary for a POD analysis to evaluate the performance of the method presented in this article, relative to existing ones. For the POD analysis, the “Hit and Miss” (HM-POD) model has been employed, following the MIL-HDBK-1823A standard. This procedure is considered the state of the art for conducting POD studies in many industries^23^, with a validated implementation in R programming language, used in the current analysis, developed by Annis^24^. The simplest POD analysis can be expressed as the ratio of true positives (TP), regarding the total number of defects, denoted as $POD=TP/(TP+FN)$, with the false negatives (FN), representing the undetected defects. This oversimplified scalar, does not reflect the real detectability related to the size or length of the defects, denoted as *a*. In many applications, the appraisal of an inspection technique is typically associated with the bigger defect to miss, and for this reason, the POD is generally expressed as a function of the defect size, defined as an ideal binary step function. In a realistic situation, the POD is typically described as an ascending sigmoid function, that can be modelled in many ways. The most accepted HM-POD model to fit the set of binary points associated to the experiments, is the log-logistic distribution^25^, written as:

6 $$\begin{aligned} POD(a)=\frac{e^{\frac{\pi }{\sqrt{3}}\left( \frac{\ln {a-m}}{\sigma }\right) }}{1+e^{\frac{\pi }{\sqrt{3}}\left( \frac{\ln {a-m}}{\sigma }\right) }} \end{aligned}$$

with *m* and $\sigma$ being the mean and standard deviation respectively. Additionally, the regression of that model has an associated uncertainty determined by a confidence interval (CI), typically estimated with the likelihood-ratio method^26^.

In this scenario, the natural cracks arising from the TIG and Laser welds have been produced on two types of alloys, namely, Inconel 718 and Haynes 282. Since the materials have different types of cracks, as well as ferromagnetic properties and surface emissivity, affecting the perceived thermal response of the signal, it has been estimated that the POD analysis must be separated in two sets with their own distinct results. A set of 13 samples with 218 cracks for the Inconel case, and 14 samples with 337 cracks for the Haynes case, has been used to conduct both analyses according to^27^, stating that a set of 60 cracks is the minimum required. The followed HM-POD, for both Haynes and Inconel sets, has consisted in the following steps.

- An enhanced FPI to stablish ground-truth and labelling of the defects and length.
- Phase-shifted induction thermography with a manual labelling of the butterfly pattern present in each phase image.
- The processing pipeline shown in Fig. 10, with the local maxima search, generating the key points overlayed in the vorticity.
- Alignment and correlation of detected points with the ground-truth.

**Figure 15:**
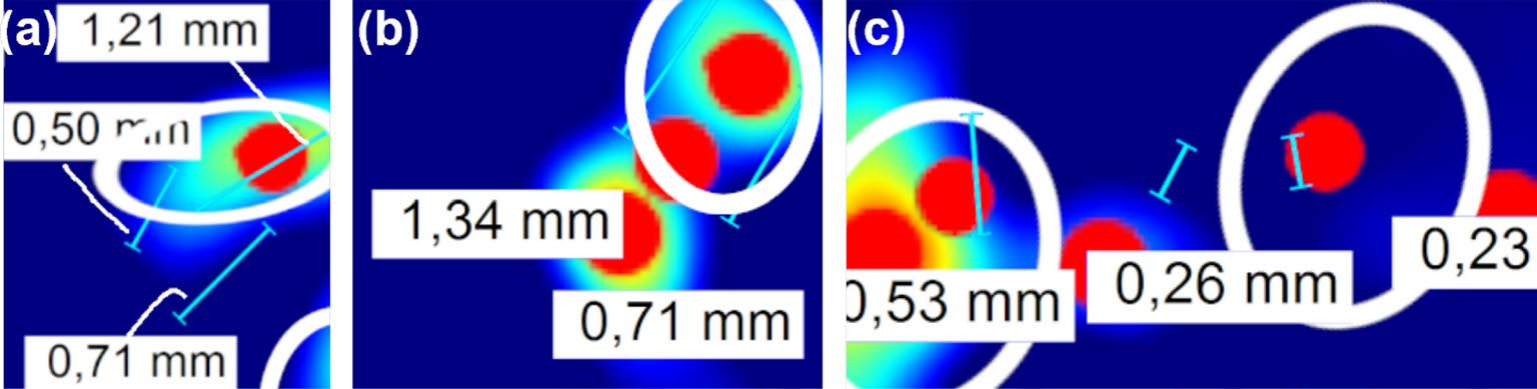
Clustered cracks association examples. Vorticity image fragments displaying an overlay of the real cracks in bright blue and the corresponding size, the detected peak of the vorticity in red, and the association of the crack and the peaks highlighted in white, yielding a number of true and false positives, as well as false negatives. (**a**) Tp = 2: 0.5, 1.21 mm, Fn = 1: 0.71 mm, Fp = 0, (**b**) Tp = 2: 1.34, 0.71 mm, (**c**) Tp = 2 0.53, 0.23 mm, Fp = 0, Fn = 1: 0.26 mm, Fp: 2.

Note that an added difficulty to the correlation of the detected points is the clustered distribution of some cracks, with a corresponding local maxima plateau covering multiple cracks, as it can be seen in Figs. 13 and 14a. The criteria that have been used to correlate the local maxima of the vorticity and the FPI ground truth has consisted in an association based on its proximity. Figure 15a shows an instance of 3 cracks and one local maxima, coinciding with 2 cracks. Figure 15b spots 2 parallel cracks matched by a set of 3 points. Figure 15c has 3 cracks, with a false negative.

Both the Inconel and Haynes have a set of 4 hit and miss values corresponding to the manual labelling of the butterfly patterns for each direction, a 5th one associated to the union of the 4 phase images, and the 6th one linked to the phase-shifting. Since the manually labelled mono-directional phase-images are so sensible to the orientation, they generate a hit-miss distribution that is very difficult to reliably fit resulting in very poor results.

**Figure 16:**
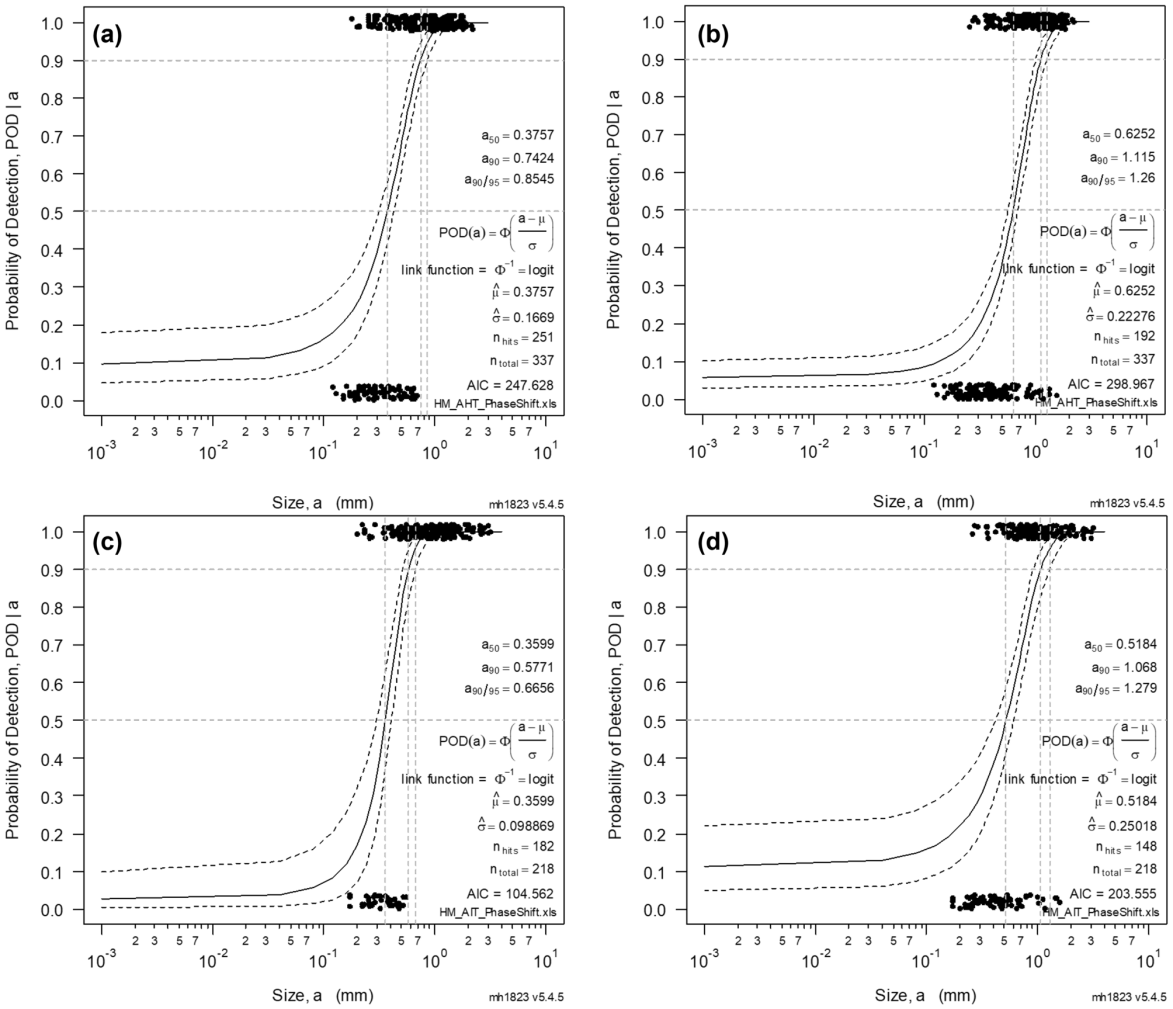
Hit/Miss Probability of Detection. Estimated POD sigmoid with a solid line, and the upper and lower confidence level with a dotted line. Four PODs corresponding to both sets and methods. (**a**) Haynes Phase-shift, (**b**) Haynes union manual labelling, (**c**) Inconel Phase-shift, (**d**) Inconel union manual labelling.

To have a fair comparison, the union of the manual labelling of all the directions has been considered to effectively employ the same input as the phase-shifted method. The HM-POD curves are shown in Fig. 16, with the upper row displaying the Haynes phase-shifted (a), and manual labelling (b). The lower row shows the TIG Inconel 718, with the phase-shifted POD (c) and its associated manual labelling (d). Note that the employed regression model has consisted in the log-logistic function implemented in the software made by Annis^24^. The corresponding results of the analysis are summarized in Table 3. As it can be observed, the resulting POD analysis in both sets is significantly more robust employing the phase-shifted. It is also worth noting that some of the identified cracks were imperceptible in the static mono-directional phase images, but their rotational component has contributed to its automatic identification.

## Conclusions

In this paper, a novel multi-directional thermo-inductive system has been proposed which is able to generate thermographies with a discrete set of predominant eddy current directions. The multiple orientations of the eddy currents generate a varying path around the defect, resulting in a thermal response dependent on a directional component. The 4 directional observations produced by the exposed system enable the detection of cracks with an unknown orientation.

The variation of the phase image pattern, regarding the magnetic field orientation, exhibits a new type of rotating feature that has not been described before. This differentiating feature has motivated the development of a processing technique, inspired by the phase-shifting, based on the integration of the relative motion of the thermal response. This directional thermal response has also been observed in a set of different samples and types of cracks.

Note that the resulting thermal response of the pulses is based on the composition of the current and precedent ones. The separation of both effects could generate an improved thermal contrast, compared to a FFT of the raw thermogram, such as the one employed in this article for simplicity. The superposition of multiple induction modalities, combining discrete or continuous directional and temporal moneodulations remains an interesting topic, which could potentially yield to complementary information.

The whole processing technique has exploited the rotating motion of the cracks in steel and superalloys such as Inconel 718, and Haynes 282, showing a strong correlation in all the exposed cases. The performance evaluation of this technique has been conducted with a ‘Hit/Miss’ Probability of Detection model, showing an increased sensitivity regarding the manual labelling of the phase-images butterfly patterns of the cracks. Future works should focus on the generalization of the technique to all types of defects, employing machine learning models, or neural networks, that would be able to carry out a fused training of the set of the discrete set of directional images. Multi-spectral and color-based Convolutional Neural Networks seem like good candidates to generalize the technique to enable an increased sensitivity of the phase-shifted induction thermography. Alternatively, the development of taylor made techniques that would focus on a sparse statistical reduction of the optical flow, into a set of trainable descriptors would also be a worthwhile option to explore in upcoming iterations. Ultimately, a rigorous physical modelling of the observed phenomena is necessary to further optimize the technique or develop new methods, as well as an eventual extrapolation to other use-cases.

**Table 3:** Hit/Miss Probability of Detection summary of both sets of welds comparing the manual labelling with the exposed method.

Set	Method	a50	a90	a90/95	nTot	nHits
		mm	mm	mm		
Haynes 282	Phase-Shift	0.3757	0.7424	0.8545	337	251
Haynes 282	Manual Labelling	0.6252	1.115	1.26	337	192
Inconel 718	Phase-Shift	0.3599	0.5771	0.6656	218	182
Inconel 718	Manual Labelling	0.5184	1.068	1.279	218	148

$a_{50}, a_{90} \text { and } a_{90/95}$ are the minimum lengths providing a probability of detection of 50 %, 90 % and 90 % with a confidence level of 95 %, respectively.

## Data Availability

The datasets used and/or analysed during the current study available from the corresponding author on reasonable request.
